# Using a theory-informed approach to guide the initial development of a post-tuberculosis care package in British Columbia, Canada

**DOI:** 10.1186/s12913-023-09835-4

**Published:** 2023-07-27

**Authors:** Kamila Romanowski, Victoria Jane Cook, Mark Gilbert, James Cameron Johnston

**Affiliations:** 1 Provincial Tuberculosis Services, British Columbia Centre for Disease Control, Vancouver, BC Canada; 2 Department of Medicine, University of British Columbia, Vancouver, Canada; 3 Clinical Prevention Services, British Columbia Centre for Disease Control, Vancouver, BC Canada; 4 School of Population and Public Health, Faculty of Medicine, The University of British Columbia, Vancouver, BC Canada

**Keywords:** Tuberculosis, Post-tuberculosis care, Behaviour change, Intervention

## Abstract

**Background:**

The importance of addressing the long-term needs of tuberculosis (TB) survivors is gaining increasing attention. One promising approach to improving post-TB care is implementing a post-TB care package. With a specific focus on the perspectives of healthcare providers in British Columbia, Canada, this study aimed to (1) determine a set of components to be included in a post-TB care package, (2) explore barriers and facilitators influencing their implementation, and (3) propose potential solutions to overcome identified challenges.

**Methods:**

Employing a multi-method approach guided by the Theoretical Domains Framework, we first conducted virtual workshops with TB care providers and utilized a modified Delphi process to establish a preliminary list of care package components. Then, we surveyed healthcare providers using closed-ended, Likert-scale questions to identify implementation barriers and enablers. Lastly, we mapped the identified barriers and enablers to establish behaviour change techniques to identify possible solutions to overcome the challenges identified.

**Results:**

Eleven participants attended virtual workshops, and 23 of 51 (45.1%) healthcare providers completed questionnaires. Identified components of the post-TB care package included:

1. Linking people with TB to a primary care provider if they do not have one.

2. Referring people with pulmonary TB for an end-of-treatment chest x-ray and pulmonary function testing.

3. Referring people with TB who smoke to a smoking cessation specialist.

4. Sharing a one-page post-TB information sheet with the patient's primary care provider, including a summary of post-TB health concerns, complications, and recommendations to prioritize age-appropriate screening for cardiovascular disease, lung cancer, and depression.

Survey results indicated that domain scores for ‘environment, context, and resources’ were the lowest, suggesting potential implementation barriers. Care navigation services to help individuals overcome health system barriers while transitioning from TB care, information leaflets, and checklists summarizing key post-TB health concerns for patients and healthcare providers to help facilitate discussions may help overcome the identified barriers.

**Conclusion:**

Healthcare providers in British Columbia acknowledge that post-TB care is integral to comprehensive health care but are limited by time and resources. Care navigation services, a post-TB checklist, and patient information leaflets may help resolve some of these barriers.

**Supplementary Information:**

The online version contains supplementary material available at 10.1186/s12913-023-09835-4.

## Contributions to the literature

- We used a theory-informed approach to guide the initial development of a post-tuberculosis care package in British Columbia, Canada
- Our findings highlight that healthcare providers in British Columbia believe post-tuberculosis care should consist of a few measures targeting key health concerns, but its implementation may be limited by time and resources.
- The methodology and findings from our study may serve as an example for other tuberculosis programs to generate actionable information to help guide the design and implementation of post-tuberculosis services.

## Background

Approximately 10 million people worldwide develop active tuberculosis (TB) every year, and about 85% of people diagnosed are successfully treated [1]. While successful treatment is often seen as a return to pre-TB health, accumulating evidence highlights that many people who survive TB face elevated mortality risk and ongoing health challenges, including pulmonary impairment, cardiovascular disease, depression, and reduced health-related quality of life [2–6]. Despite the recognition of these long-term needs, TB programs have traditionally focused on timely diagnosis, treatment adherence and prevention, with limited attention given to post-TB care.

Nevertheless, the importance of addressing the long-term needs of TB survivors is gaining increasing attention [7]. Although limited evidence exists to identify effective interventions addressing the health challenges faced by TB survivors [8], newly published international standards and guidelines, including the 2022 Canadian TB Standards, emphasize the importance of assessing and managing post-TB complications [9, 10]. Recommendations include assessment for post-TB lung disease, pulmonary function testing for people with pulmonary TB, and addressing comorbidities during TB treatment in partnership with primary care providers [10]. However, the optimal strategies for implementing these recommendations remain unclear.

Implementing care packages is a promising approach to improving post-TB care and addressing comorbidities. Care packages typically comprise of three to five evidence-informed interventions or practices performed collectively and reliably [11]. Often, they are considered complex interventions due to their multifaceted nature and the need for behaviour change among those responsible for their implementation; thus, using a theoretical approach when developing care packages can enhance the understanding of healthcare providers’ behaviours and facilitate successful design and implementation [12].

The Theoretical Domains Framework (TDF) is a widely used theoretical framework that encompasses 14 domains of behaviour change [13, 14]. It was developed using an expert consensus process and validation to identify psychological and organizational theories relevant to health practitioner clinical behaviour change [13, 14]. The process of using the TDF for intervention design has been outlined in detail and applied extensively [13, 15–17].

In this study, we employed a multi-method approach, utilizing the TDF to guide the initial development of a post-TB care package in British Columbia, Canada. With a specific focus on the perspectives of healthcare providers, we aimed to (1) determine the components to be included in a post-TB care package, (2) explore barriers and facilitators influencing their implementation, and (3) propose potential solutions to overcome identified challenges.

## Methods

### Study setting

This research was conducted in British Columbia, Canada, a province with 4.9 million people and an active TB incidence of 6.0 per 100 000 population [18], in conjunction with Provincial TB Services at the British Columbia Centre for Disease Control (BCCDC). The BCCDCProvincial TB Services is a centralized health agency responsible for diagnosing and treating most people with active and latent TB throughout British Columbia; they also consult with healthcare providers and coordinate case management for drug-resistant and complex pediatric TB. Embedded within TB services are two provincial TB clinics located in Greater Vancouver.

Ethical approval of the study was provided by the University of British Columbia Behavioural Research Ethics Board (Behavioural Research Ethics Board Certificate #H22-00732). We report this study following the Guidance for Reporting Intervention Development Studies in Health Research (GUIDED) checklist (Supplementary Appendix) [19].

### Study design

**Fig. 1:**
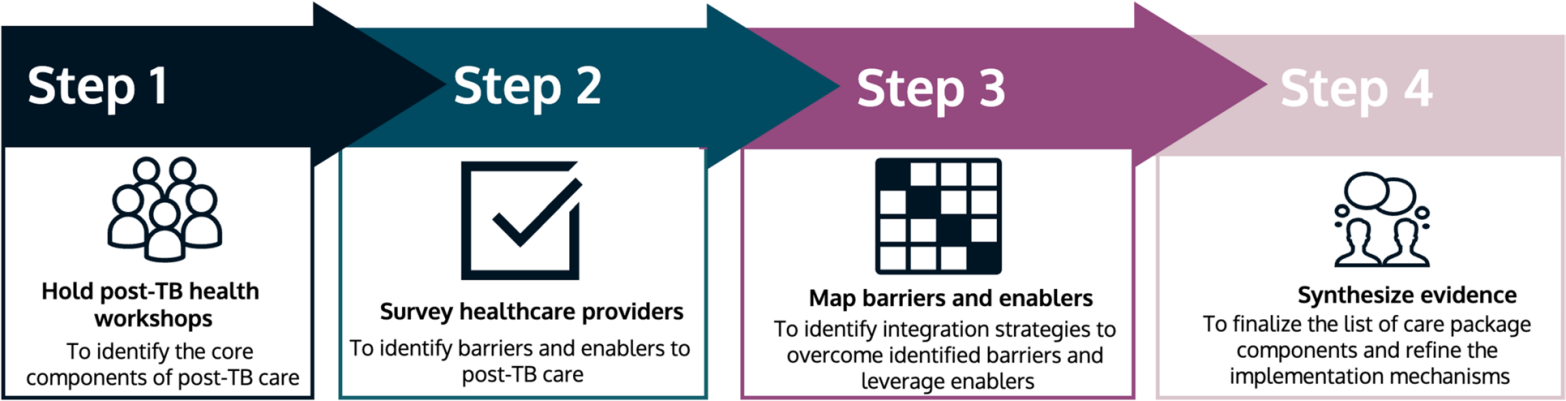
Overview of the development process

For this study, we followed the steps outlined by French et al. on developing theory-informed interventions using the TDF. [13] Briefly, these steps include (1) determining the target behaviours, (2) exploring what enablers and facilitators need to be addressed for successful implementation, (3) identifying possible solutions to overcome the barriers and facilitators, and (4) synthesizing the evidence to design the intervention package. For each step, we worked collaboratively with healthcare providers and relevant stakeholders [20]. The process we used is illustrated in Fig. 1, and each step is described below.

### Step 1: Post-TB care workshops

First, we held a series of virtual workshops with healthcare providers specializing in TB care at the BCCDC-TB clinics and general practitioners in British Columbia to determine a preliminary list of components to be included in a post-TB care package. Using convenience sampling from urban and rural areas, we recruited TB healthcare providers and general practitioners. At these workshops, we presented participants with an overview of the evidence from a scoping review on existing interventions to reduce post-TB morbidity [21] and national and international standards on post-TB care [9, 10]. During these workshops, we discussed post-TB care and potential post-TB care package components (Supplemental Table 1), focusing on their applicability in TB clinics in British Columbia.

We then used a modified Delphi consensus method to identify which components should be considered key components of post-TB care package in British Columbia [22]. We used an anonymous online voting platform to reach a consensus on a preliminary list of components to be included in a post-TB care package. All participants had the opportunity to add any other potential components they believed were missing before voting began.

The online voting platform included a disclaimer which stood in place of a signed formal consent form. Participants needed to agree to the disclaimer before participating in the voting. Each potential post-TB care package component was presented and then rated as “agree,” “disagree,” or “neither agree nor disagree.” When consensus on a component was reached, we summarized it in behavioural terms, including target behaviour, the content to be delivered, to whom, by what mode, and how often [23].

### Step 2: Survey healthcare providers

#### Survey healthcare providers

Once we determined a preliminary list of components to include in a post-TB care package, we surveyed healthcare providers working within the BCCDC-TB clinics to identify any barriers and enablers to their successful implementation.

#### Questionnaire development

We developed an anonymous, self-administered, web-based questionnaire for assessing the domains of behaviour change. The questionnaire was adapted from a generic English questionnaire provided by Huijg et al. (2014), which was developed to discriminately assess the majority of TDF domains and can be tailored to suit different contexts [24].

The final version of the questionnaire consisted of 22 items (two to five items per domain). It covered eight TDF domains: knowledge, skills, professional role, beliefs about capabilities, consequences, optimism, environment, and memory. We excluded behaviour regulation, goals, and reinforcement, as prior research shows these domains lack discriminant validity [24, 25]. We also excluded the domain's social influence, intention, and emotion, as initial feedback indicated that our initial survey required tailoring to increase acceptance. A copy of the questions and the corresponding domain for each question is presented in Supplemental Table 2. The questions were randomly arranged, and respondents were asked to respond on a five-point Likert scale, from strongly agree to disagree strongly.

We also included two questions about which components of post-TB care healthcare providers believed were acceptable and which components they believed were part of their role as healthcare providers.

#### Participants and data collection

All healthcare providers (nursing, physician, pharmacist, and social workers) at the BCCDC-TB clinics were invited to participate via email between May and June 2022. Participants meeting the inclusion criteria were sent an invitation email, which explained the purpose of the study, estimated completion time, and a link to the survey. The survey was conducted using a web-based Qualtrics Survey Tool. The beginning of the survey had a disclaimer which stood in place of a signed formal consent form. Participants needed to agree to the disclaimer before participating in the survey. We sent a reminder email two and four weeks after the initial invitation, and the survey was championed at internal staff meetings.

#### Statistical analysis

Returned surveys were given a sequential identification number. We calculated internal consistency estimates for each theoretical domain using Cronbach’s alpha, with a cut-off of 0.50, as used in prior preliminary intervention design research [16, 17]. An alpha of less than 0.50 indicated variability in how healthcare providers responded to the different questions within that domain. Individual domain scores were based on responses measured on a five-point Likert scale (1 = strongly disagree, 5 = strongly agree); the scales were reversed for negatively worded questions. We calculated a mean domain score and standard deviation for each domain. The domain scores were then presented; a low mean domain score suggests the domain may be a barrier to implementation, while a high value indicates the domain may facilitate implementation. All analyses were conducted in R (V.4.0.2.) [26].

### Step 3: Map barriers and enablers

Once survey results were compiled, we mapped the identified barriers and enablers to appropriate behaviour change techniques to identify implementation mechanisms. This was done using the Theory and Techniques tool [27]. The Theory and Techniques Tool is a rigorously developed matrix to help identify integration strategies to overcome barriers and leverage enablers identified in Step 2 [27–29]. It is based on synthesized evidence from the literature and expert consensus on individual strategies' effectiveness in eliciting behaviour change [27–29].

### Step 4: Evidence synthesis

After synthesizing all available evidence, study team members and implementation partners, including healthcare providers and administrators within BCCDC-Provincial TB Services, virtually reviewed the evidence to refine the components and implementation mechanisms. Implementation partners were invited to participate via email and selected based on their relevant expertise. Discussion points focused on what was locally appropriate, feasible, and could be implemented as part of a cohesive approach to post-TB care in British Columbia. Discussions also encompassed potential outcome measures that could be used to evaluate the effectiveness of the care package once it has been implemented.

## Results

### Step 1: Post-TB health workshops

Eight participants attended our first workshop, including three TB physicians, four TB nurses, and one social worker working within the TB Clinics. Three general practitioners participated in our second workshop. Identified post-TB care package components are presented in Table 1 and include (1) linking people to a primary care provider, (2) referring people with pulmonary TB for an end-of-treatment chest x-ray and pulmonary function testing, (3) referring people who smoke to a smoking cessation specialist and (4) providing an information sheet to the patient's primary care provider on post-TB health.

**Table 1:** Potential post-TB care package components identified during the healthcare provider workshops (Step 1)

Target behaviour	Target population	Who performs the behaviour
Discuss post-TB health	People with pulmonary and non-pulmonary TB	TB nurse, TB doctor
Link to primary care provider	People with pulmonary and non-pulmonary TB	Social worker
Refer to a smoking cessation specialist	People with pulmonary and non-pulmonary TB	TB nurse, TB doctor
Refer for an end of treatment chest x-ray	People with pulmonary TB	TB doctor
Refer for end of treatment pulmonary function testing	People with pulmonary TB	TB doctor
Administer end-of-treatment 6-min walk test	People with pulmonary TB	TB nurse
Send primary care provider a post-TB care information sheet	People with pulmonary and non-pulmonary TB	TB administrative staff

Discussions during the workshop with general practitioners emphasized the need for care navigation services for individuals to help them overcome healthcare system barriers and facilitate timely access to quality healthcare while transitioning out of TB care. Additional concerns raised by general practitioners were appropriate and timely transitions to primary care and the need for increased communication between TB healthcare providers and general practitioners rather than the need to screen for multiple comorbidities in this population.

### Step 2: Survey healthcare providers

A total of 23 of 51 questionnaires (45.1%) were completed and returned. The distribution of responses is presented in Supplemental Table 2, and the demographic characteristics of questionnaire participants are shown in Supplemental Table 3. Internal consistency for each theoretical domain and the mean domain score are presented in Table 2. The mean domain scores were highest for optimism, knowledge, and professional role. Conversely, beliefs about consequences, capabilities, and environment, context, resources had the lowest mean domain scores.

**Table 2:** Mean domain scores and Cronbach’s alpha for the healthcare provider survey (Step 2)

Domain	Domain description within context of research	Number of questions used to measure each domain	Cronbach’s alpha	Mean domain score (SD)
Optimism	Are healthcare providers optimistic that by providing post-TB care desired goals will be obtained?	5	0.78	4.24 (1.04)
Knowledge	Are healthcare providers aware of the long-term impacts of TB?	3	0.58	3.86 (1.26)
Professional role	Do healthcare providers feel that it is part of the professional role to provide post-TB care?	4	0.54	3.54 (1.25)
Skills	Do healthcare providers feel they have the correct training to provide post-TB care?	2	0.92	3.50 (1.38)
Beliefs about capabilities	Do healthcare providers feel confident and comfortable providing post-TB care?	2	0.57	3.35 (1.33)
Beliefs about consequences	What do healthcare providers think will happen as a result of providing post-TB care? What do they see as a benefit of this work?	2	0.56	3.00 (1.01)
Memory, attention, decision process	Do healthcare providers remember to discuss post-TB care?	2	0.19^a^	3.11 (1.32)
Environment, context, resources	Do healthcare providers have enough resources to provide post-TB care?	3	0.70	2.51 (1.35)

^a^The included questions may not reliably address the constructs of the domain. Results are presented for this domain; however, no conclusions are drawn from them

We identified seven key barriers to implementing post-TB care, including healthcare providers not having enough time, handouts, or referral options to discuss post-TB health concerns; them not feeling confident providing post-TB care; and being more concerned about other TB issues than post-TB health concerns. Facilitators included respondents believing it is their responsibility as care providers to discuss post-TB health concerns as part of routine care, and that incorporating post-TB care as routine care would help improve the long-term health of this population (Table 3).

**Table 3:** Identified barriers mapped to the theoretical domains and corresponding behaviour change techniques (Step 3)

Barriers and facilitators identified	TDF	Corresponding BCT	Potential operationalized component
HCPs do not have enough time to discuss post-TB health	Environment, context, resources	Restructure the environment	Add an additional 5 min to the last clinic appointment
HCPs may not be trained to provide post-TB health recommendations	Skills	Instructions on how to perform behaviours	Routine educational meetings on post-TB health for healthcare providers
HCPs may not feel confident providing post-TB heath	Skills	Instructions on how to perform behaviours	Routine educational meetings on post-TB health for healthcare providers
HCPs may not be aware of the evidence that supports incorporating post-TB care	Knowledge	Information about health consequences	Information leaflets for healthcare providers which summarize key post-TB health concerns and evidence on incorporating post-TB care
HCPs do not have enough handouts to discuss post-TB health	Environment, context, resources	Add objects to the environment	Information leaflets for patients which summarize key post-TB health concerns
HCPs do not have enough referral options to discuss post-TB health	Environment, context, resources	Add objects to the environment	Identify post-TB ‘system navigators’ to coordinate the referral process
HCPs may be more concerned about other TB issues than post-TB health	Consequences	Information about health consequences	Information leaflets for healthcare providers which summarize key post-TB health concerns and evidence on incorporating post-TB care
HCPs believe that incorporating post-TB care as routine care would improve the long-term health of this population	Optimism	No modifiable BCTs	
HCPs believe it is their role as care providers to discuss post-TB health concerns as part of routine care	Professional role	No modifiable BCTs	

*Abbreviations*: *TDF* Theoretical Domains Framework, *BCT* Behaviour Change Technique, *HCPs* Healthcare Providers, *TB* Tuberculosis

Our results also indicated that over 70% of respondents supported linking patients to primary healthcare providers, referring those who smoke to a smoking cessation specialist, and assessing for post-TB lung disease. However, only approximately 60% of respondents believed that it was part of their role as healthcare providers to perform these actions (Supplemental Fig. 1).

### Step 3: Map barriers and enablers

We then mapped the identified barriers and enablers to at least one theoretical domain and corresponding behaviour change techniques (Table 3). As there are no conclusive links between the domain’s optimism and professional role and modifiable behaviour change techniques, these were not included in our final mapping process [27–29]. The corresponding behaviour change techniques to overcome the barriers identified included restricting and adding objects to the environment, providing instructions on how to perform behaviours, and providing information about health consequences [27]. Potential operationalized components to overcome these barriers include information leaflets for healthcare providers which summarize key post-TB health concerns and evidence on incorporating post-TB care, routine educational meetings on post-TB health for healthcare providers and identify post-TB system navigators to coordinate the referral process (Table 3).

### Step 4: Evidence synthesis

A review of evidence from Steps 1, 2, and 3 suggests that healthcare providers in British Columbia believe post-TB care should be a simple and systematic process, including the following components:

1. Routinely linking people with TB to a primary care provider if they do not have one
2. Routinely referring people with pulmonary TB for an end-of-treatment chest x-ray and pulmonary function testing
3. Routinely referring people with TB who smoke to a smoking cessation specialist
4. Routinely sending a one-page post-TB information sheet to the patient's primary care provider. This information sheet should include a summary of post-TB health concerns, complications, and recommendations to consider age-appropriate screening for cardiovascular disease, lung cancer, and depression.

**Fig. 2:**
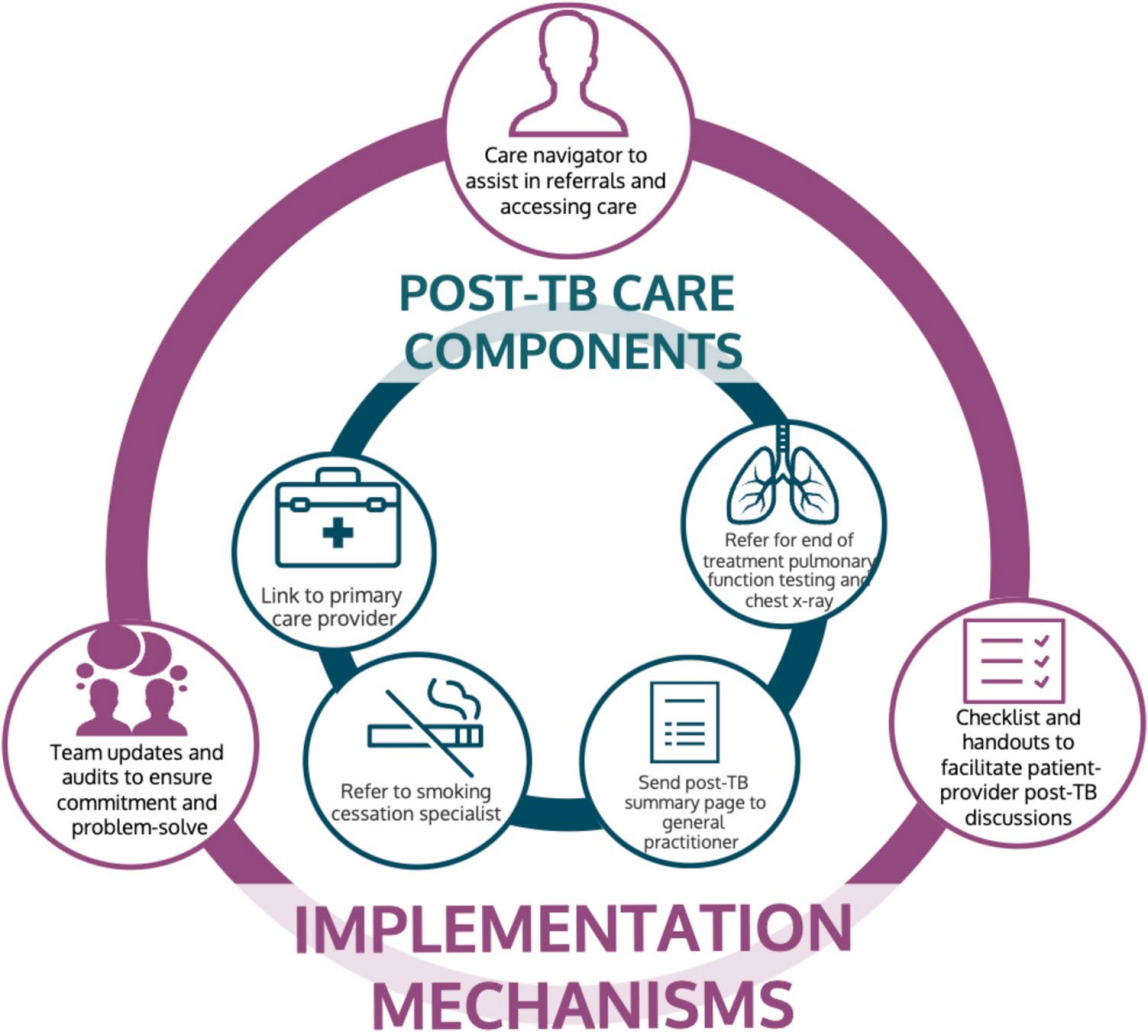
Proposed mechanism for delivering post-TB care in British Columbia

To implement these post-TB care components, care navigators should be identified to help coordinate and facilitate access to care. Additionally, information leaflets and checklists for patients and healthcare providers summarizing key post-TB health concerns need to be created to help facilitate discussions. Lastly, routine post-TB team updates and audits are necessary to ensure commitment to post-TB care implementation and provide an opportunity to problem-solve. Key suggested outcome measures included the proportion of patients linked to a primary care provider and the proportion of patients who completed post-treatment chest X-ray and pulmonary function testing. A proposed mechanism for delivering post-TB care based on these findings is presented in Fig. 2.

## Discussion

Results from our study highlight that healthcare providers in British Columbia, Canada believe a post-TB care package should encompass a few measures targeting key healthcare concerns, including (1) routinely linking people with TB to a primary care provider if they do not have one, (2) referring people with pulmonary TB for an end-of-treatment chest x-ray and pulmonary function testing, (3) referring people with TB who smoke to a smoking cessation specialist, and (4) sending a one-page post-TB information sheet to the patient's primary care provider. Our findings also showcase the challenges healthcare providers face in delivering post-TB care, particularly time and resource constraints.

To address these barriers, we propose care navigation services to help streamline and support healthcare providers in providing post-TB care. Additionally, creating physician checklists and culturally appropriate post-TB information leaflets can help facilitate post-TB health discussions between providers and patients. Lastly, we recommend routine post-TB team updates and audits to provide an opportunity to problem-solve and ensure uptake. These proposed solutions were grounded in prior literature linking behaviour change techniques to the TDF domains [27], and then refined based on was is locally appropriate, feasible, and could potentially be implemented as part of a cohesive approach to post-TB care in British Columbia.

One key theme from discussions during the virtual workshops was the importance of care navigation services to help bridge the service gap for populations completing treatment and transitioning out of TB care. Navigation programs were initially developed to address inequitable access to cancer care but have since expanded to provide more holistic person-centred care and to identify and resolve patients' barriers to care [30]. In Canada, the burden of TB is not shared equally; foreign-born individuals and Canadian-born Indigenous peoples continue to be disproportionately affected by TB [31]. These populations are historically underserved and often face barriers to accessing care and navigating a structurally racist healthcare system [32]. Transitions between various healthcare services are potential points for fragmented care, which can be complicated for patients and caregivers [33]. These challenges are further compounded for older adults with multiple comorbidities, new migrants whose primary language is not the language care is provided, or people whom the healthcare system has historically mistreated [32]. Thus, we believe care navigation services should be considered a key component of TB care in British Columbia.

This study demonstrates that leveraging the TDF and a robust multi-method approach can help bridge the gap between post-TB care recommendations and their implementation. The methodology and findings can provide a valuable resource for researchers and practitioners seeking to adopt and implement post-TB care strategies. Moreover, the systematic and iterative process presented in this study can be adapted to various contexts and settings and applied to address similar gaps in adopting and implementing guidelines for other health interventions*.*

### Strengths and limitations

A key strength of our study is its theoretical basis and systematic presentation of post-TB care development in British Columbia. A further strength was our multidisciplinary, collaborative approach, and we ensured that healthcare providers specializing in TB care were involved in different steps. We used workshops with healthcare providers to identify the potential components of post-TB care, and we surveyed healthcare providers to identify barriers and enablers specific to the British Columbia context. Lastly, our research team was multidisciplinary, blending expertise in TB care, primary care, research, and implementation science.

Limitations included a risk of selection bias, as our sample of participants likely consists of highly engaged healthcare providers who already have knowledge of post-TB care. This may have led to underestimating the barriers encountered in post-TB care. Furthermore, with only 23 of 51 questionnaires returned, the limited response rate highlights sample size constraints. The low response rate may have been due to a lack of an honorarium or paid time set aside to participate. Nevertheless, the questionnaire respondents included a diverse group of TB healthcare providers. Thus, we are confident we covered a broad range of relevant aspects.

Our questionnaire results also exhibited relatively low internal consistency, with four of the eight scores demonstrating a Cronbach’s alpha below 0.7. This suggests a moderate ability of the questionnaire to measure the underlying TDF domains accurately. A low internal constituency can compromise the accuracy and validity of the survey results and may also limit our ability to draw reliable conclusions from the survey data. Thus, our survey results should be interpreted with caution. We also only engaged with healthcare providers in British Columbia. Therefore, our findings may only be generalizable to other high-resource, low-TB incidence regions. However, the theory-informed, systematic process we used to obtain our results can be easily adapted to various settings so they can determine locally relevant approaches to post-TB care.

Additionally, the recommended interventions assume there is the capacity for them to take place. In Canada, access to primary care is challenging, with approximately 18% of British Columbians reporting they do not have access to a regular healthcare provider [34, 35]. Thus, a lack of available services, particularly in under-served rural communities, may make it challenging to link TB survivors to primary care providers. Moving forward, we plan to examine the implementation and feasibility of the identified components of post-TB care, including cost-effectiveness data. Given the resource constraints TB programs face, it will also be essential to engage with policymakers and funders of care for strategic alignment and resource allocation for post-TB care.

Lastly, excluding people diagnosed with TB and their caregivers is a significant limitation of our work. Involving people with lived experience can lead to interventions that are more responsive to their needs, as affected communities offer valuable insights into the pressing challenges they face and the optimal approach to addressing them [36, 37]. Thus, the next stage of this research is to explore perceptions of post-TB care among people diagnosed with TB and their caregivers, including the acceptability and uptake of the proposed interventions.

## Conclusion

By systematically applying theory and collaborating with team members and knowledge users, we identified healthcare providers specializing in TB care who agree with the need to provide post-TB care as part of comprehensive care in British Columbia. However, a perceived lack of time and resources influences their ability to offer post-TB care effectively. Our results indicate that healthcare providers in British Columbia believe post-TB care should be a simple and systematic process supported by trained care navigators. While post-TB care is complex and multifaceted, our results recommend a practical guide to developing and implementing post-TB care in British Columbia.

## Supplementary Information

**Additional file 1. **GUIDED – a guideline for reporting for intervention development studies.

**Additional file 2: Supplemental Table 1.** Potential post-TB care package components presented during workshops with healthcare providers (Step 1). **Supplemental Table 2.** Healthcare provider survey questions, their corresponding domain, and the distribution of responses (Step 2). **Supplemental Table 3.** Demographic characteristics of the healthcare provider survey participants (Step 2). **Supplemental Figure 1.** Beliefs on which components should be incorporated as part of the post-TB care from the healthcare provider survey (Step 2).

## Data Availability

The datasets used and/or analyzed during the current study are available from the corresponding author upon reasonable request.

## Abbreviations

TB: Tuberculosis
TDF: Theoretical Domains Framework
BCCDC: British Columbia Centre for Disease Control
