# Supplementary material for: Using a theory-informed approach to guide the initial development of a post-tuberculosis care package in British Columbia, Canada
Source: BMC Health Serv Res. 2023 Jul 27;23:805. doi: 10.1186/s12913-023-09835-4 (PMC10375626; doi:10.1186/s12913-023-09835-4)
Supplement: Supplementary file 2 — Additional file 2: Supplemental Table 1. Potential post-TB care package components presented during workshops with healthcare providers (Step 1). Supplemental Table 2. Healthcare provider survey questions, their corresponding domain, and the distribution of responses (Step 2). Supplemental Table 3. Demographic characteristics of the healthcare provider survey participants (Step 2). Supplemental Figure 1. Beliefs on which components should be incorporated as part of the post-TB care from the healthcare provider survey (Step 2). [file 12913_2023_9835_MOESM2_ESM.pdf]

Supplemental Table 1. Potential post-TB care package components presented during workshops with healthcare providers (Step 1)

Category	Target population	Component
**Screening for post-TB lung disease**	People with pulmonary TB	Spirometry<br>Full pulmonary function testing<br>6-minute walk test<br>Chest x-ray<br>CT Scan<br>Borg Dyspnea Scale<br>Cough severity visual analog scale
**Linkage to primary care practitioner**	People with pulmonary and non-pulmonary TB	Ask if person has routine care provider<br>Provide resources to link to care if they do not<br>Ensure clinic actively identifies a primary care practitioner
**Smoking cessation**	People with pulmonary and non-pulmonary TB	Ask if person smokes<br>Give brief advice on smoking cessation<br>Offer smoking cessation support<br>Refer to smoking cessation specialist
**Cardiovascular risk assessment**	People with pulmonary and non-pulmonary TB	Blood pressure screening<br>Lipid screening<br>Framingham (or similar) CVD screening<br>Inform family practitioner/routine care provider of results to help transition care
**Comorbidity screening**	People with pulmonary and non-pulmonary TB	Confirm Hep B & C screening completed<br>Confirm DM screening completed<br>Confirm HIV screening completed
**Depression screening**	People with pulmonary and non-pulmonary TB	Administer Patient Health Questionnaire -9 (or similar), and inform family practitioner of results
**Substance use disorder screening**	People with pulmonary and non-pulmonary TB	Screen for substance use disorder and inform family practitioner of results
**Vaccinations**	People with pulmonary and non-pulmonary TB	Assess if person is up to date on current vaccinations (COVID, flu, pneumococcal)<br>Offer COVID vaccination<br>Offer flu vaccination<br>Offer pneumococcal vaccination
**Other**		

Supplemental Table 2. Healthcare provider survey questions, their corresponding domain, and the distribution of responses (Step 2)

	Domain	Strongly agree	Somewhat agree	Neither agree nor disagree	Somewhat disagree	Strongly disagree
I am aware of the long-term impacts of TB	Knowledge	15 (65.2)	8 (34.8)	0 (0.0)	0 (0.0)	0 (0.0)
Incorporating post-TB care as part of routine care for people with active TB would help improve the long-term health of this population	Optimism	16 (69.6)	7 (30.4)	0 (0.0)	0 (0.0)	0 (0.0)
I believe it is my responsibility to discuss post-TB health with patients who have active TB	Professional role	18 (78.3)	4 (17.4)	0 (0.0)	0 (0.0)	1 (4.3)
I am trained to provide post-TB health recommendations to people who have active TB	Skills	5 (21.7)	8 (34.8)	4 (17.4)	4 (17.4)	2 (8.7)
I feel confident discussing post-TB health with people who have active TB	Capabilities/ Skills	9 (39.1)	5 (21.7)	2 (8.7)	4 (17.4)	3 (13.0)
Other topics take priority for me over post-TB health discussions	Professional role	3 (13.0)	8 (34.8)	8 (34.8)	2 (8.7)	2 (8.7)
I worry that discussing post-TB health may increase anxiety in people who have active TB	Consequences	1 (4.3)	6 (26.1)	10 (45.3)	4 (17.4)	2 (8.7)
I am aware of the evidence that supports incorporating post-TB care as part of routine care[+]	Knowledge	6 (26.1)	7 (30.4)	4 (17.4)	4 (17.4)	1 (4.3)
I don't typically discuss post-TB health with patients because I forget	Memory/ attention	1 (4.3)	5 (21.7)	5 (21.7)	6 (26.1)	6 (26.1)
I don't typically discuss post-TB health with patients because I don't know what to recommend	Memory/ attention	5 (21.7)	6 (26.1)	4 (17.4)	6 (26.1)	2 (8.7)
I currently have enough time to discuss post-TB health with patients	Environment/ Resources	3 (13.0)	5 (21.7)	6 (26.1)	2 (8.7)	7 (30.4)

I have enough handouts to discuss post-TB health with patients	Environment/ Resources	1 (4.3)	0 (0.0)	5 (21.7)	9 (39.1)	8 (34.8)
I have enough referral options to discuss post-TB health with patients	Environment/ Resources	3 (13.0)	5 (21.7)	5 (21.7)	3 (13.0)	7 (30.4)
My colleagues are supportive of incorporating post-TB care as part of routine TB care	Professional role	3 (13.0)	6 (26.1)	11 (47.8)	2 (8.7)	1 (4.3)
I am familiar with the new Canadian TB Standards guidelines regarding post-TB health	Knowledge	4 (17.4)	12 (52.2)	1 (4.3)	3 (13.0)	3 (13.0)
I am more concerned about other TB related issues than post-TB health+	Consequences	1 (4.3)	7 (30.4)	8 (34.8)	5 (21.7)	2 (8.7)
I feel optimistic that if I provide advice on post-TB health, patients will follow it	Capabilities	5 (21.7)	9 (39.1)	7 (30.4)	2 (8.7)	0 (0.0)
I only discuss post-TB health with patients who ask about it	Professional role	0 (0.0)	6 (26.1)	9 (39.1)	3 (13.0)	5 (21.7)
I believe that it is beneficial for patients completing treatment for active TB to be: a. Screened for post-TB respiratory disease+	Optimism	12 (52.2)	8 (34.8)	2 (8.7)	0 (0.0)	0 (0.0)
b. Screened for cardiovascular disease+	Optimism	8 (34.8)	11 (47.8)	3 (13.0)	0 (0.0)	0 (0.0)
c. Screened for depression+	Optimism	9 (39.1)	9 (39.1)	4 (17.4)	0 (0.0)	0 (0.0)
d. Linked to a primary healthcare provider+	Optimism	19 (82.6)	3 (13.0)	0 (0.0)	0 (0.0)	0 (0.0)

+ Missing one response

Supplemental Table 3. Demographic characteristics of the healthcare provider survey participants (Step 2)

Characteristic	N (%)
**Current occupation**	
MD	11 (47.8)
Nurse	9 (39.1)
Pharmacist	2 (8.7)
Administrator	1 (4.3)
**Years as a healthcare provider**	
< 1	0 (0.0)
1 – 5	2 (8.7)
6 -10	5 (21.7)
> 10	16 (69.6)

Supplemental Figure 1. Beliefs on which components should be incorporated as part of the post-TB care from the healthcare provider survey (Step 2)

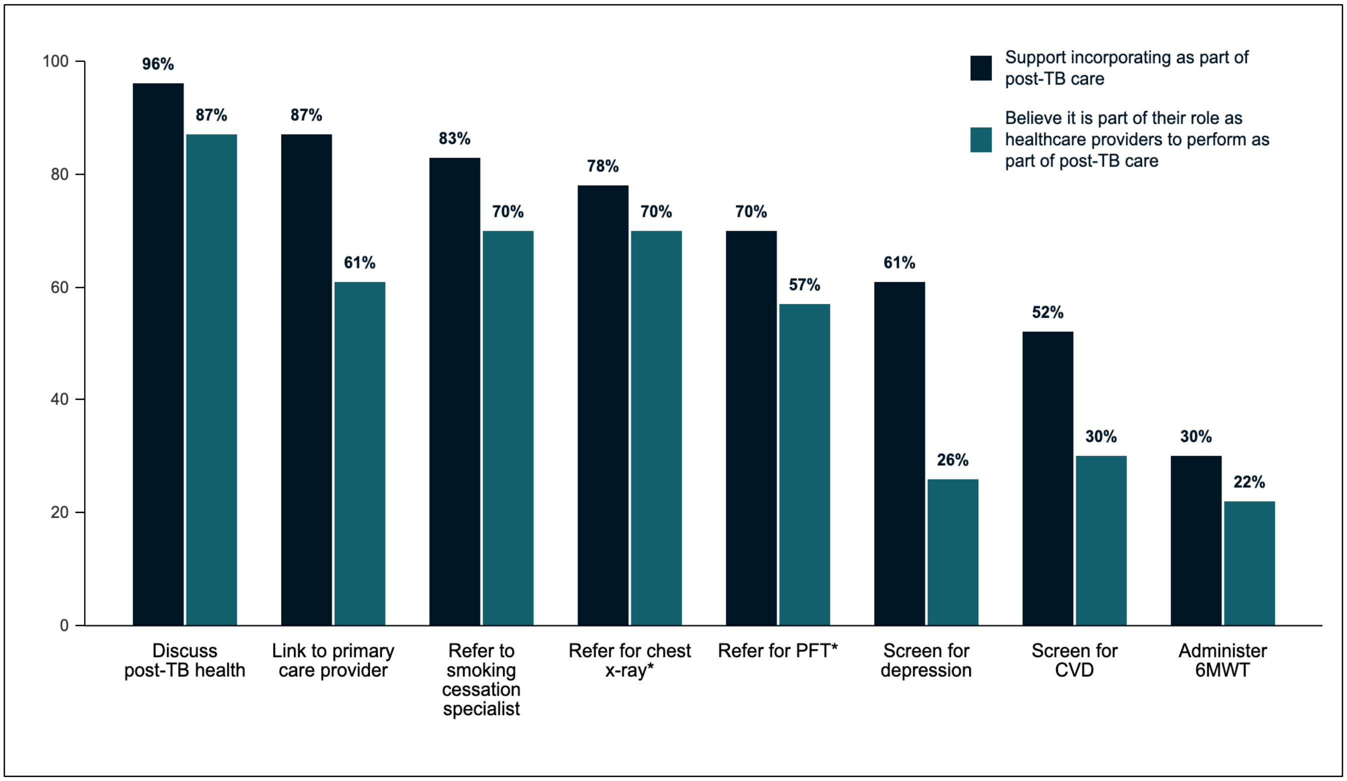

*For people with pulmonary TB. PFT = pulmonary function testing; 6MWT = 6-minute walk test; CVD = cardiovascular disease
